# Illinois State Medical Society

**Published:** 1873-05-01

**Authors:** T. D. Fitch

**Affiliations:** Secretary; Chicago


Secretary's Office,
Illinois State Medical Society,
Chicago, April 10, 1873.

The twenty-third annual session of the Society will be held in the city of Bloomington, Ill., May 20, 1873, at 10 a.m.

The following committees are expected to report:

STANDING COMMITTEES.

*Practical Medicine*—S. K. Crawford, M.D., Monmouth; D. L. Jewett, M.D., Watseka; H. M. Lyman, M.D., Chicago.

*Surgery*—J. L. White, M.D., Bloomington; J. B. Hamilton, M.D., Kane; H. W. Kendall, M.D., Quincy.

*Ophthalmology* — E. L. Holmes, M.D., Chicago; J. P. Johnson, M.D., Peoria; F. C. Hotz, M.D., Chicago.

*Otology*—S. J. Jones, M.D., Chicago; Chas. Hunt, M.D., Dixon; T. Galt, M.D., Rock Isl'd.

*Obstetrics*—T. D. Fitch, M.D., Chicago; A. Niles, M.D., Quincy; D. S. Jenks, M.D., Plano.

*Drugs and Medicines* — J. H. Hollister, M.D., Chicago; E. P. Cook, M.D., Mendota; J. L. Hamilton, M.D., Peoria.

*Necrology*—G. W. Albin, M.D., Neoga; J. O. Hamilton, M.D., Jerseyville; J. K. Secord, M.D., Elmwood.

SPECIAL COMMITTEES.

*Galvano. Therap.*—D. Prince, M.D., Jacksonville.

*Hernia*—J. H. Reeder, M.D., Lacon.

*Hygiene*—T. F. Worrell, M.D., Bloomington.

*On the Assistance Necessary and Justifiable in Difficult and Protracted Labors*—S. B. Hawley, M.D., Aurora.

*Stricture of the Urethra*—John Murphy, M.D., Peoria.

*Phthisis Pulmonalis*—H. A. Johnson, M.D., Chicago.

*Phys. Nerv. Syst.* — C. W. Earle, M.D., Chicago.

*Cerebro-Spinal Meningitis* — G. Wheeler Jones, Danville.

*Locomotor Ataxia* — N. S. Davis, M.D., Chicago.

*Orthopedic Surgery*—J. S. Sherman, M.D., Chicago.

*To Prepare and Deliver a Public Address at the next Meeting of the Society*—Geo. T. Allen, M.D., Springfield.

T. D. FITCH, M.D., *Secretary*.

